# Mediator Preference of Two Different FAD-Dependent Glucose Dehydrogenases Employed in Disposable Enzyme Glucose Sensors

**DOI:** 10.3390/s17112636

**Published:** 2017-11-16

**Authors:** Noya Loew, Wakako Tsugawa, Daichi Nagae, Katsuhiro Kojima, Koji Sode

**Affiliations:** 1 Department of Biotechnology and Life Science, Graduate School of Engineering, Tokyo University of Agriculture and Technology, 2-24-16 Naka-cho, Koganei, Tokyo 184-8588, Japan; noyaloew@cc.tuat.ac.jp (N.L.); tsugawa@cc.tuat.ac.jp (W.T.); sode-lab@cc.tuat.ac.jp (D.N.); 2 Institute of Global Innovation Research, Tokyo University of Agriculture and Technology, 3-8-1 Harumi-cho, Fuchu, Tokyo 183-8538, Japan; 3 Ultizyme International Ltd., 1-13-16, Minami, Meguro, Tokyo 152-0013, Japan; kojima@ultizyme.jp

**Keywords:** glucose dehydrogenase, flavin adenine dinucleotide, *Aspergillus flavus*, *Burkholderia cepacia*, enzyme sensor strip, mediated electron transfer, potassium ferricyanide, methoxy phenazine methosulfate, hexaammineruthenium chloride

## Abstract

Most commercially available electrochemical enzyme sensor strips for the measurement of blood glucose use an artificial electron mediator to transfer electrons from the active side of the enzyme to the electrode. One mediator recently gaining attention for commercial sensor strips is hexaammineruthenium(III) chloride. In this study, we investigate and compare the preference of enzyme electrodes with two different FAD-dependent glucose dehydrogenases (FADGDHs) for the mediators hexaammineruthenium(III) chloride, potassium ferricyanide (the most common mediator in commercial sensor strips), and methoxy phenazine methosulfate (mPMS). One FADGDH is a monomeric fungal enzyme, and the other a hetero-trimeric bacterial enzyme. With the latter, which contains a heme-subunit facilitating the electron transfer, similar response currents are obtained with hexaammineruthenium(III), ferricyanide, and mPMS (6.8 µA, 7.5 µA, and 6.4 µA, respectively, for 10 mM glucose). With the fungal FADGDH, similar response currents are obtained with the negatively charged ferricyanide and the uncharged mPMS (5.9 µA and 6.7 µA, respectively, for 10 mM glucose), however, no response current is obtained with hexaammineruthenium(III), which has a strong positive charge. These results show that access of even very small mediators with strong charges to a buried active center can be almost completely blocked by the protein.

## 1. Introduction

The market for glucose sensing devices used for glycemic level control is ever expanding due to the increasing number of people suffering from diabetes mellitus worldwide. Because more and more patients regularly check their blood glucose levels themselves to apply the right treatment, the demand for simple yet accurate devices for the self-monitoring of blood glucose (SMBG) is growing. Current commercially available sensors for SMBG are, for the most part, second-generation electrochemical biosensors. In this type of biosensors, the analyte (glucose) is oxidized by an enzyme (glucose recognition element). Then, the cofactor of the enzyme is reduced. Then, the reduced cofactor reduces an artificial electron acceptor (mediator). This mediator then is re-oxidized at the electrode, which generates a response current.

An ideal electron mediator should: (a) React rapidly with the reduced enzyme, (b) have reversible electron transfer kinetics with a low overpotential at least for the oxidation at the electrode, which should be independent of pH, (c) have stable oxidized and reduced forms, and (d) should not react with oxygen [1].

One of the most commonly used mediators in commercial SMBG-type sensors and enzyme electrode research is ferricyanide (oxidized form). Both the oxidized and the reduced (ferrocyanide) form are easily available, stable in dry and dark conditions, water soluble, small, inorganic complexes with a strong negative charge. The ferro-/ferricyanide redox couple shows reversible electron transfer kinetics and is often used as a standard redox probe for electrochemical characterization of electrodes. However, enzyme sensor strips with potassium ferricyanide require special packaging to ensure dry and dark storage conditions.

Recently, hexaammineruthenium(III) chloride has been gaining attention for the use as mediator in commercial sensor strips. Hexaammineruthenium chloride is easily available, sufficiently stable in both oxidized (Ru^III^) and reduced (Ru^II^) form, soluble in water and positively charged in both forms. The hexaammineruthenium(II/III) redox couple has a lower redox potential than the ferro-/ferricyanide redox couple, so that enzyme sensor strips with hexaammineruthenium(III) chloride can be used at lower operating potentials than enzyme sensor strips with potassium ferricyanide. In general, a lower operating potential leads to fewer interferences. Furthermore, the packaging requirements for enzyme sensor strips with hexaammineruthenium(III) chloride are less stringent than those for strips with potassium ferricyanide. Therefore, hexaammineruthenium(III) chloride is one of the most attractive mediators for SMBG-type sensor strips. However, reports on the construction of glucose sensors with hexaammineruthenium(III) chloride are rare.

Among the two enzymes commonly used as glucose recognition element, glucose oxidase (GOx) and glucose dehydrogenase (GDH), the latter is more suited for second generation sensors, because GDH does not react with oxygen [2].

Among the different types of GDHs, those harboring flavin adenine dinucleotide (FAD) as cofactor are diverse in structure and characteristics depending on their origin. Bacterial FAD-dependent glucose dehydrogenase (FADGDH), derived from *Burkholderia cepacia*, is a thermostable, hetero-trimeric enzyme complex consisting of a catalytic subunit containing FAD in its redox center (*α* subunit), a multi-heme cytochrome *c*-type electron transfer subunit (*β* subunit), and a small chaperone-like subunit (*γ* subunit) [3]. We previously developed SMBG-type glucose sensors based on bacterial FADGDH with hexaammineruthenium(III) chloride as mediator [4,5].

Fungal FADGDH, e.g., derived from *Aspergillus flavus*, is a monomeric enzyme and in structure similar to fungal GOx, which also has FAD as a cofactor [2,6,7]. Both native (with glycosylation) and recombinant (without glycosylation) fungal FADGDHs have a narrow substrate specificity compared to other GDHs and specifically do not show activity towards maltose [6,8]. Therefore, fungal FADGDHs are especially attractive enzymes for use in glucose sensing.

Although many have attempted to fine-tune mediators to be used with FADGDHs, e.g., osmium complexes [9] or quinone derivatives [10,11], the investigated mediators are mostly organic compounds or metal complexes with organic ligands. Tsuruoka et al., recently conducted a more systematic comparison of organic electron acceptors of fungal FADGDH [12]. However, to the best of our knowledge, there is no report comparing mediators often used in commercial sensor strips, such as ferricyanide and hexaammineruthenium(III).

Furthermore, although there are reports comparing various fungal FADGDHs [13], there is no report directly comparing fungal and bacterial FADGDHs. Although the oxidation of glucose occurs at a FAD in both cases, and thus can be expected to have similar characteristics, the transfer of electrons to the electron acceptor occurs with very different mechanisms, suggesting differing characteristics in their preferences for electron acceptors.

In this report, we compare enzyme electrodes with combinations of bacterial and fungal FADGDH with three different mediators, potassium ferricyanide, hexaammineruthenium(III) chloride and methoxy phenazine methosulfate (mPMS; electroactive form of phenazine methosulfate, which is often used in spectrophotometric assays), two of which are used in various commercial sensor strips for the measurement of blood glucose. With this comparison, we aim to achieve a better understanding about what characterizes a suitable mediator and how this is influenced by the structure and electron transfer mechanism of the enzyme. Furthermore, we hope to establish the investigation of the mediator preference of enzymes used in SMBG sensor strips as an important sub-section of the research on the improvement of blood glucose measurements.

## 2. Materials and Methods

### 2.1. Materials, Enzymes and Electrodes

Phenazine methosulfate (PMS), methoxy phenazine methosulfate (mPMS), 2,6-dichlorophenolindophenol (DCIP), and potassium hexacyanoferrate(III) (K_3_[Fe(CN)_6_], ferricyanide) were obtained from Kanto Chemical Co. Inc. (Tokyo, Japan). Hexaammineruthenium(III) chloride ([Ru(NH_3_)_6_]Cl_3_) was purchased from Sigma-Aldrich (St. Louis, MO, USA). All other chemicals were of analytical grade.

Bacterial FADGDH, FADGDH*γαβ*(QY), was prepared according to [14]. This enzyme, derived from *Burkholderia cepacia*, was engineered to contain the point mutations Ser326Gln and Ser365Tyr. Briefly, *Escherichia coli* BL21 was co-transformed with the expression vector for FADGDH*γαβ*(QY) and the vector encoding genes needed for cytochrome c maturation. The transformed cells were grown in LB medium containing 50 μg/mL ampicillin and 50 μg/mL kanamycin. Harvested cells were resuspended in 20 mM Tris-HCl (pH 7.5), containing 0.1% Triton X-100, and lysed by sonication. The solubilized membrane fraction was applied to anion exchange chromatography and gel filtration. Thus purified enzyme (V_max_ = 160 U/mg, K_m_ = 3.2 mM with glucose) was suspended in 10 mM 3-morpholinopropane-1-sulfonic acid (MOPS) buffer (pH 7.0), containing 0.1% Triton X-100.

Fungal FADGDH, *Af*GDH, derived from *Aspergillus flavus*, was prepared according to [7]. Briefly, *E. coli* BL21 (DE3) was transformed with the expression vector for *Af*GDH. The transformed cells were grown in LB medium containing 30 μg/mL kanamycin. Harvested cells were resuspended in 10 potassium phosphate buffer (PPB, pH 6.5), and lysed using a French press. The soluble fraction was purified by anion exchange chromatography. Thus purified enzyme (V_max_ = 330 U/mg, K_m_ = 16 mM with glucose) was suspended in 10 mM MOPS (pH 7.0).

Disposable screen-printed carbon electrode (SPCE)-strips with two carbon-plate electrodes and an Ag/AgCl electrode were obtained from i-SENS Inc. (Seoul, Korea). The working electrode had an area of 2.4 mm^2^.

### 2.2. Activity Assay

Enzyme activity was determined by mixing a sample of the respective enzyme with PMS or mPMS (final concentration 0.6 mM) and DCIP (final concentration 0.06 mM) in 10 mM MOPS buffer (pH 7.0) and, to start the reaction, glucose, xylose, or maltose (various concentrations) and monitoring the decrease of DCIP absorbance at 600 nm. The reduction of 1 µmol DCIP in 1 min, corresponding to the oxidation of 1 µmol/min glucose, was defined as 1 U dehydrogenase activity. All measurements were carried out in triplicate.

### 2.3. Electrochemical Measurements

To fabricate the enzyme electrodes, 0.09 U (determined with 5 mM glucose and mPMS/DCIP) FADGDH*γαβ*(QY) or *Af*GDH (0.83 µg, or 1.3 µg, respectively) in 1 µL 10 mM MOPS (pH 7.4) was dried onto the electrode area of SPCE-strips at 4 °C. Then, a spacer and cover were attached to each electrode to form a capillary space.

For the electrochemical measurements, samples containing 0–40 mM substrate and 100 mM mediator were prepared. As substrate, glucose, maltose, xylose, or mixtures of glucose and maltose or glucose and xylose was used. As mediator, mPMS, potassium ferricyanide, or hexaammineruthenium(III) chloride was used. 1 µL sample was injected into the capillary space of the enzyme electrodes. Five seconds after loading the sample, +200 mV (in case of mPMS or hexaammineruthenium(III)) or +400 mV (in case of ferricyanide) vs. Ag/AgCl was applied and the current was recorded with a HSV-100, Hokuto Denko Co. (Tokyo, Japan). Measurements were carried out in triplicate.

## 3. Results

### 3.1. Substrate Specificity of FADGDH

Native bacterial FADGDH has a broad substrate specificity and thus is little suited for glucose test strips. We previously engineered bacterial FADGDH to improve its suitability, and reduce activity towards maltose [3]. Here, the engineered bacterial FADGDH, FADGDH*γαβ*(QY), was used. *Af*GDH was used without modification.

**Figure 1:**
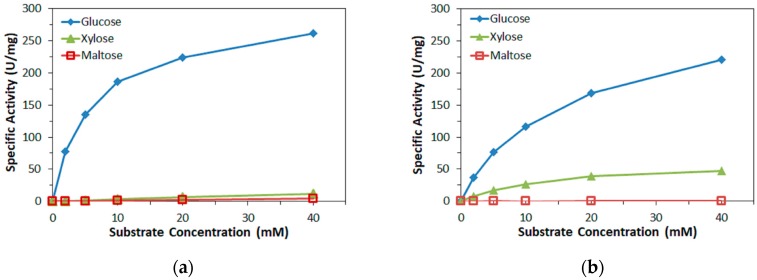
Dependency of specific activity on the concentration of three mono- and disaccharides for two FAD-dependent glucose dehydrogenases (FADGDHs). (**a**) bacterial FADGDH (FADGDH*γαβ*(QY)), (**b**) fungal FADGDH (*Af*GDH).

To confirm the substrate specificity of the FADGDHs used in this study, the specific activity of the two enzymes towards glucose, xylose, and maltose was measured with PMS as primary electron acceptor and plotted against the substrate concentration in Figure 1. The activity of *Af*GDH towards glucose was slightly lower than that of FADGDH*γαβ*(QY). Both FADGDHs are relatively specific for glucose, and showed no or minimal activity towards maltose. The activity towards xylose of *Af*GDH was significant, while that of FADGDH*γαβ*(QY) was minimal.

### 3.2. Glucose Measurements with Different Mediators

**Figure 2:**
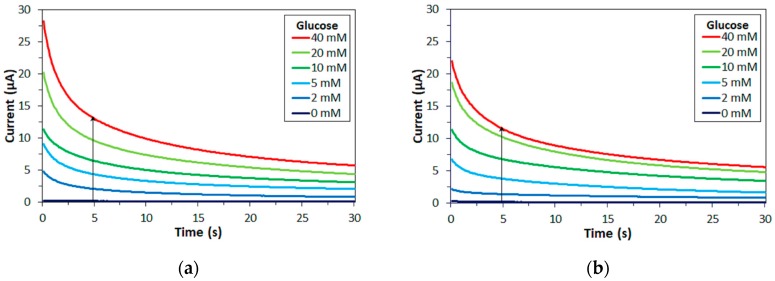
Representative time courses of glucose measurements with enzyme electrodes. Mediator: mPMS. Enzyme: (**a**) FADGDH*γαβ*(QY), (**b**) *Af*GDH (arrow: increasing glucose concentration).

Enzyme electrodes were fabricated with either FADGDH and the amount of enzyme was adjusted so that the activity towards 5 mM glucose, determined with mPMS and DCIP as electron acceptors, was the same for each electrode and enzyme. Mixtures of different glucose concentrations and 100 mM of either mPMS, ferricyanide or hexaammineruthenium(III) were loaded onto the electrodes and the current was monitored over time. Figure 2 shows time courses for measurements of different glucose concentrations for enzyme electrodes employing FADGDH*γαβ*(QY) or *Af*GDH with mPMS as mediator.

The principle of the measurements here is that of most commercial SMBG meters. In these types of measurements, the enzyme, mediator, and analyte react in a μL-scale volume. The potential is applied after the enzyme reaction reaches its equilibrium. The response current follows the Cottrell equation, and is an indicator for the concentration of the reduced mediator at equilibrium. Typically, the response current decays initially and reaches a steady plateau after several seconds [4,15,16] Corresponding time courses were recorded with ferricyanide and with hexaammineruthenium(III) as mediator.

**Figure 3:**
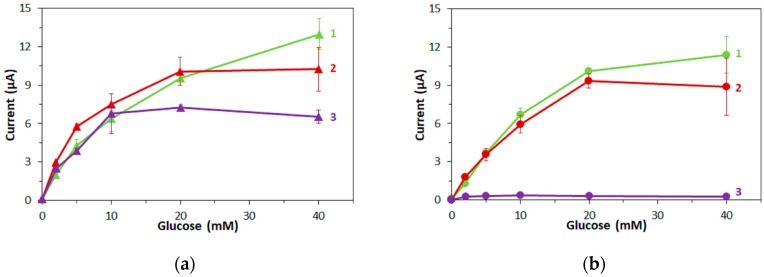
Dependency of current at 5 s after application of the potential on the glucose concentration. Enzyme: (**a**) FADGDH*γαβ*(QY), (**b**) *Af*GDH. Mediator: (1) 100 mM mPMS, (2) 100 mM ferricyanide, (3) 100 mM hexaammineruthenium(III).

In Figure 3, the response current at 5 s after application of the potential is plotted against the glucose concentration for the two types of enzyme electrodes with (1) mPMS, (2) ferricyanide and (3) hexaammineruthenium(III) as mediator.

Both types of FADGDH electrodes showed similar responses depending on the glucose concentration when mPMS or ferricyanide was used as mediator (Figure 3), especially at concentrations below 20 mM. For mPMS, this is not surprising since the enzyme activity on the electrodes was adjusted with this mediator. The response current of electrodes with FADGDH*γαβ*(QY) with hexaammineruthenium(III) as mediator was similar to that with mPMS for up to 10 mM glucose, but did not increase for higher glucose concentrations (Figure 3a). No response current was obtained with electrodes employing *Af*GDH when hexaammineruthenium(III) was used (Figure 3b).

### 3.3. Interference Due to Maltose with Different Mediators

Although maltose is not present in blood endogenously, it is used as a stabilizing agent or osmolality regulator in various medical preparations, such as some intravenous drugs. Also, peritoneal dialysis solution contains icodextrin, which is metabolized to maltose. Therefore, maltose has to be checked as interfering agent for commercial SMBG test strips. The US Food and Drug Administration recommends the testing of 10,000 mg/dL (292 mM) maltose [17]. Ideally, 95% of all SMBG results should be within ±15% of the reference measurement and 99% of all SMBG results should be within ±20%.

**Figure 4:**
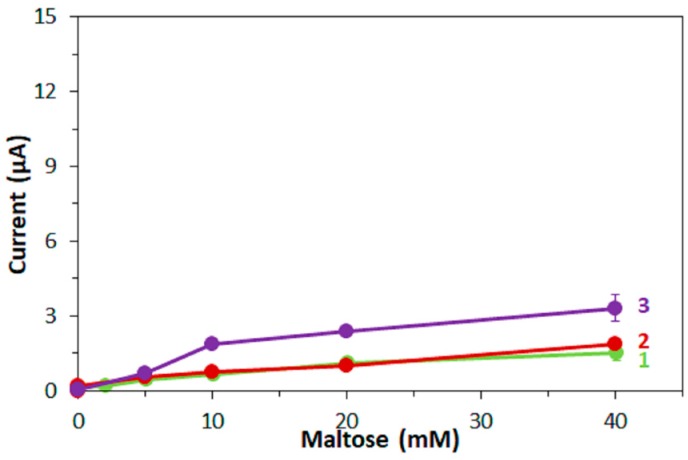
Dependency of current at 5 s after application of the potential on the maltose concentration. Enzyme: FADGDH*γαβ*(QY). Mediator: (1) 100 mM mPMS, (2) 100 mM ferricyanide, (3) 100 mM hexaammineruthenium(III).

First, corresponding measurements of maltose were carried out with the same amounts of enzyme on the electrodes as were used for the glucose measurements. The response currents are plotted against the maltose concentrations in Figure 4 for FADGDH*γαβ*(QY) as enzyme. With FADGDH*γαβ*(QY) electrodes, a low response current was obtained with mPMS and ferricyanide as mediator and a higher response current was obtained with hexaammineruthenium(III). All response currents to maltose were significantly lower than those to glucose. *Af*GDH did not show any activity towards maltose and consequently no response currents to maltose were obtained with *Af*GDH electrodes.

**Figure 5:**
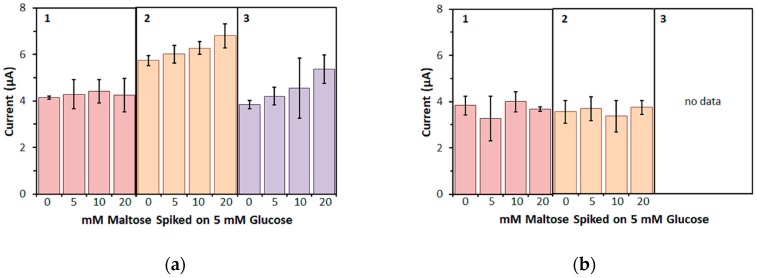
Response current to 5 mM glucose spiked with different amounts of maltose. Enzyme: (**a**) FADGDH*γαβ*(QY), (**b**) *Af*GDH. Mediator: (1) 100 mM mPMS, (2) 100 mM ferricyanide, (3) 100 mM hexaammineruthenium(III).

Next, to investigate the influence of maltose on the measurement of glucose more closely, a physiological glucose concentration (5 mM) was spiked with several concentrations of maltose (Figure 5). With FADGDH*γαβ*(QY), the bias on the response current for 5 mM glucose was negligible for up to 20 mM maltose when mPMS was used as mediator (Figure 5a). When ferricyanide was used as mediator, the bias on the response current for 5 mM glucose increased slightly with increasing maltose concentration and was +18% for 20 mM maltose. A higher bias was observed with hexaammineruthenium(III) as mediator and was +18% for 10 mM maltose and +39% for 20 mM maltose. *Af*GDH electrodes showed no bias on the response current for 5 mM glucose with both mPMS and ferricyanide (Figure 5b). Because *Af*GDH electrodes did not show any response to glucose when hexaammineruthenium(III) was used, no spiking experiments were carried out for this case.

### 3.4. Interference Due to Xylose with mPMS as Mediator

Although xylose is not present in blood endogenously either, its presence should be considered in patients subjected to a xylose absorption test, a clinical test for the evaluation of malabsorption. Therefore, xylose, too, has to be checked as interfering agent for commercial SMBG test strips. The US Food and Drug Administration recommends the testing of 200 mg/dL (13 mM) xylose [17].

**Figure 6:**
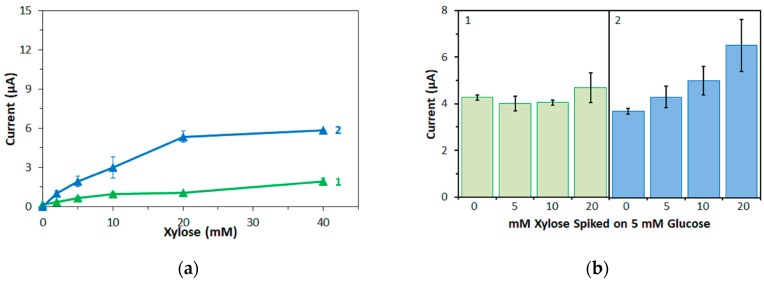
(**a**) Dependency of current at 5 s after application of the potential on the xylose concentration. (**b**) Response current to 5 mM glucose spiked with different amounts of xylose. Enzyme: (1) FADGDH*γαβ*(QY); (2) *Af*GDH. Mediator: mPMS.

First, corresponding measurements were also carried out with xylose and with mPMS as mediator, again with the same amounts of enzyme on the electrodes as were used for the glucose measurements. The response currents are plotted against the xylose concentration in Figure 6a for both enzymes. Again, to investigate the influence of xylose on the measurement of glucose, a physiological glucose concentration (5 mM) was spiked with several concentrations of xylose (Figure 6b).

FADGDH*γαβ*(QY) electrodes showed similar response currents to xylose and maltose (Figure 4 and Figure 6a). *Af*GDH electrodes showed significantly higher response currents to xylose than to maltose. For low concentrations (2 mM), the response current of *Af*GDH electrodes to xylose were similar as those to glucose, while for higher concentrations, the response currents to xylose were lower than those to glucose (Figure 3b and Figure 6a).

FADGDH*γαβ*(QY) electrodes showed a low bias on the response current for 5 mM glucose at high xylose concentrations (+10% for 20 mM xylose, Figure 6b). *Af*GDH electrodes showed a significant bias on the response current for 5 mM glucose even for low concentrations of xylose (+16%, +35%, and +76% for 5 mM, 10 mM, and 20 mM xylose, respectively; Figure 6b). The results with *Af*GDH electrodes are in agreement with our previous study [15].

## 4. Discussion

The three mediators used in this study were: (1) mPMS, (2) ferricyanide, and (3) hexaammineruthenium(III) (Table 1). Ferricyanide and hexaammineruthenium(III) are small inorganic compounds, while mPMS is an organic compound. The reduced form of mPMS is uncharged, while the oxidized form has a positive charge. Both reduced and oxidized forms of the ferro/ferricyanide couple have strong negative charges, and both reduced and oxidized forms of hexaammineruthenium(II/III) have strong positive charges. The redox potential of the ferro/ferricyanide redox couple is +0.23 V vs. Ag/AgCl [18], while mPMS and hexaammineruthenium(II/III) have similar redox potentials at −0.14 V and −0.11 V vs. Ag/AgCl, respectively [19,20].

Among the three tested mediators, mPMS and ferricyanide are suitable electron acceptors for both FADGDHs, while hexaammineruthenium(III) can accept electrons only from bacterial FADGDH, but not from fungal FADGDH. The ability to accept electrons from an enzyme should depend on the redox potential of the mediator: A high redox potential of the mediator should facilitate the electron transfer. Indeed, in a recent study on *Aspergillus terreus*-derived FADGDH (*At*GDH) and organic electron acceptors, Tsuruoka et al., concluded that the redox potential of the mediator is the main influencing factor on the electron transfer kinetics [12].

However, the redox potentials of all three mediators used in this study are within the range of redox potentials of the mediators investigated by Tsuruoka et al., which all accepted electrons from a fungal FADGDH. Therefore, the redox potential is unsuitable to explain why hexaammineruthenium(III) does not accept electrons from the fungal FADGDH.

According to Tsuruoka et al., charged groups can hinder the access of the mediator to the active center of the enzyme and thus decrease the electron transfer rate [12]. However, all mediators in [12] were organic, i.e., had hydrophobic parts, and had no or a low charge. Furthermore, Tsuruoka et al., did not distinguish between positive and negative charges and did not investigate mediators with strong charges. In contrast, two of the three tested mediators in this study, ferricyanide and hexaammineruthenium(III), had strong charges and no hydrophobic parts. In fact, they could be described as “isolated charged groups”. Furthermore, they were of opposite charges. The third mediator, mPMS, was similar to those investigated in [12], i.e., organic, with no or low charge.

The results in this study reveal that the charge of a mediator has a greater influence on the ability of enzymes to transfer electrons to this mediator than indicated in [12]. Furthermore, the sign of the charge is of great importance. In particular, fungal FADGDH tolerates strong negative charges (ferricyanide) fairly well, while it does not accept mediators with strong positive charges, and the access of hexaammineruthenium(III) to the active center of the enzyme is blocked, despite the small size of this mediator. This is the first report of a mediator being blocked from accessing the active center of an enzyme because of its charge.

Moreover, ruthenium(III) complexes are known to accept electrons from fungal FAD-dependent glucose oxidase (GOx) and pyrroloquinoline quinone-dependent GDH [22]. Hexaammineruthenium(III) chloride is also used as mediator in some commercial SMBG test strips utilizing GOx as glucose recognition element. Because of the structural similarity of fungal FAD-dependent GDH and fungal FAD-dependent GOx, the fact that fungal FADGDH cannot utilize hexaammineruthenium(III) as electron acceptor, while GOx can, is rather surprising and requires further, more detailed investigations.

Furthermore, while in fungal FADGDH, electrons are transferred directly from the FAD to the mediator, in bacterial FADGDH, electrons are transferred from the FAD via a 3Fe-4S-type iron-sulfur-cluster to a multi-heme *c* subunit before they are transferred to the mediator [23]. Therefore, considering only the redox potential of the mediator, bacterial FADGDH should accept a smaller range of mediators. However, bacterial FADGDH did transfer electrons to all three tested mediators. The slight difference between ferricyanide and hexaammineruthenium(III) can, in this case, be explained by the redox potentials of the two mediators. Thus, for bacterial FADGDH, the charge of the mediator has a low influence and the ability to transfer electrons to a mediator is determined by the redox potential of the mediator.

In fact, bacterial FADGDH is capable of transferring electrons directly to an electrode and we have been exploiting this for direct electron transfer type glucose sensors and for enzyme fuel cells [14,24,25,26]. It seems that the multi-heme *c* subunit does not distinguish the charge or size and structure of the electron acceptor and thus can transfer electrons to any electron acceptor as long as it is electrochemically possible.

Furthermore, in this study, the bias on the glucose response current due to maltose was also investigated with the three mediators. For enzyme sensor strips with bacterial FADGDH, the relative bias due to maltose depended on the mediator. A higher bias due to maltose was observed when hexaammineruthenium(III) was used as mediator compared to the bias when ferricyanide was used. With mPMS as mediator, no bias in presence of maltose was observed. Enzyme sensor strips with fungal FADGDH did not show any response current for maltose and therefore no bias due to maltose was observed. Therefore, the bias of enzyme sensor strips due to interferences seems to depend on the mediator.

The electron acceptor preference of glucose oxidase has been engineered away from molecular oxygen and towards artificial electron acceptors [27,28,29]. Similar approaches, such as random mutagenesis or rational design based on structural studies, might lead to an engineered fungal FADGDH that is capable of transferring electrons to hexaammineruthenium(III). SMBG test strips consisting of such an engineered fungal FADGDH and hexaammineruthenium(III) chloride should have very low interferences, due to the substrate specificity of fungal FADGDH and the low operating potential made possible by the mediator. Such test strips should also have less stringent packing requirements, which should lead to lower production costs.

## 5. Conclusions

In this study, the preferences of enzyme sensor strips with two different FADGDHs for mediators with a strong positive, a strong negative or a low charge were investigated and compared. Both FADGDHs preferred the mediator with low charge (mPMS). The fungal FADGDH tolerated negative charges (ferricyanide), while it did not accept positive charges (hexaammineruthenium(III)). Bacterial FADGDH, which contains a subunit facilitating the electron transfer, tolerated both positive and negative charges. These results show that even very small electron acceptors cannot pass freely through the enzyme scaffold to a buried active center and access of mediators with strong charges can be almost completely blocked by the protein. Structural investigations of the enzymes might reveal one or more pathways for electron acceptors with aligning amino acid residues influencing the “mediator selectivity”.

Furthermore, the influence of the mediator on the response current of enzyme sensor strips to major and minor enzyme substrates might differ. Therefore, the bias of enzyme sensor strips due to interferences has to be investigated separately for each new mediator.

Of the three mediators investigated in this study, hexaammineruthenium(III) chloride is the most desirable for application in commercial SMBG test strips; the low redox potential allows for a low operating potential, thus reducing electrochemical interferences, and the higher storage stability allows for less stringent packing requirements compared to strips containing potassium ferricyanide. However, this study revealed that hexaammineruthenium(III) cannot be used as primary electron acceptor for one of the most desirable enzymes for use in commercial SMBG test strips, fungal FADGDH.

The random mutagenesis or rational design based on structural studies, might lead to an engineered fungal FADGDH that is capable of transferring electrons to hexaammineruthenium(III), and consequently realize SMBG test strips consisting of such an engineered fungal FADGDH and hexaammineruthenium(III) chloride, showing very low interferences, due to the substrate specificity of fungal FADGDH and the low operating potential made possible by the mediator.

## Figures and Tables

**Table 1:** Properties of primary electron acceptors in this study.

Electron Acceptor	Structure/Formula	Redox Potential ^1^	Charge ^2^	Method ^3^
PMS	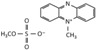	−0.09 V [21]	+/0	activity measurement
mPMS	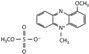	−0.14 V [19]	+/0	enzyme sensor
Hexaammineruthenium(III)	[Ru(NH_3_)_6_]Cl_3_	−0.11 V [20]	+3/+2	enzyme sensor
Ferricyanide	K_3_[Fe(CN)_6_]	+0.23 V [18]	−3/−4	enzyme sensor

^1^ vs. Ag/AgCl. ^2^ oxidized form/reduced form. ^3^ Main method the electron acceptor was used in this study.
